# The Association Between Heat-Shock Protein Polymorphisms and Prognosis in Lung Cancer Patients Treated With Platinum-Based Chemotherapy

**DOI:** 10.3389/fphar.2020.01029

**Published:** 2020-07-21

**Authors:** Ting Zou, Jun-Yan Liu, Li She, Ji-Ye Yin, Xi Li, Xiang-Ping Li, Hong-Hao Zhou, Juan Chen, Zhao-Qian Liu

**Affiliations:** ^1^National Institution of Drug Clinical Trial, Xiangya Hospital, Central South University, Changsha, China; ^2^Department of Clinical Pharmacology, Hunan Key Laboratory of Pharmacogenetics, Xiangya Hospital, Central South University, Changsha, China; ^3^National Clinical Research Center for Geriatric Disorders, Xiangya Hospital, Central South University, Changsha, China; ^4^Department of Orthopaedics, The First Affiliated Hospital of the University of South China, Hengyang, China; ^5^Department of Otolaryngology Head and Neck Surgery, Xiangya Hospital, Central South University, Changsha, China; ^6^Otolaryngology Major Disease Research Key Laboratory of Hunan Province, Xiangya Hospital, Central South University, Changsha, China; ^7^Department of Pharmacy, Xiangya Hospital, Central South University, Changsha, China

**Keywords:** lung cancer, platinum-based chemotherapy, prognosis, genetic polymorphism, heat shock proteins

## Abstract

**Objective:**

Lung cancer is one of the most prevalent cancers and the leading cause of cancer-related death in the world. Platinum-based chemotherapy plays an important role in lung cancer treatment, but the therapeutic effect varies from person to person. Heat shock proteins (HSPs) have been reported to be associated with the survival time of lung cancer patients, which may be a potential biomarker in lung cancer treatment. The aim of this study was to investigate the association between genetic polymorphisms and the prognosis in lung cancer patients treated with platinum-based chemotherapy.

**Methods:**

We performed genotyping in 19 single nucleotide polymorphisms (SNPs) of HSP genes and Rho family genes of 346 lung cancer patients by SequenomMassARRAY. We used Cox proportional hazard models, state and plink to analyze the associations between SNPs and the prognosis of lung cancer patients.

**Results:**

We found that the polymorphisms of *HSPB1* rs2070804 and *HSPA4* rs3088225 were significantly associated with lung cancer survival (*p*=0.015, *p*=0.049*, respectively). We also discovered the statistically significant differences between rs2070804 with age, gender, histology and stage, rs3088225 with gender and stage, which can affect lung cancer prognosis.

**Conclusion:**

The results of our study suggest that *HSPB1* rs2070804 (G>T) and *HSPA4* rs3088225 (A>G) may be useful biomarkers for predicting the prognosis of lung cancer patients treated with platinum-based chemotherapy.

## Introduction

Lung cancer is one of the deadliest malignancies in the world (Siegel et al., 2019). It can be divided into small cell lung cancer (SCLC) and non-small cell lung cancer (NSCLC). NSCLC consists of adenocarcinoma, squamous cell cancer and large cell lung cancer (Granger, 2016;  The Lancet Respiratory, 2017). The main treatments of lung cancer are surgery, radiotherapy, chemotherapy and immunotherapy, and platinum-based chemotherapy is the first-line chemotherapy regimens (Dunbar et al., 2018). However, most of the patients are at advanced stage when diagnosed (Woodman et al., 2020). The ongoing treatments are limited by drug-resistance and unpredictable adverse-drug-reactions (ADR) (Hong et al., 2020; Sun et al., 2020). They are big challenges preventing clinical therapeutic benefits, which lead to a very low five-year survival rate (Clausen & Langer, 2019). In recent years, more and more investigation found that the therapy effect varies with different individuals (Li et al., 2019; Zou et al., 2019). There are many gene polymorphisms that have been found to be associated with drug resistance or adverse drug reactions in lung cancer patients treated with platinum-based chemotherapy, such as translation initiation factor 3a (*eIF3a*) (Xu et al., 2013), Wnt-inducible signaling pathway protein 1 (*WISP1*) (Chen et al., 2015), DNA repair genes (*XRCC5, RRM1*) (Chen et al., 2016; Zheng et al., 2017), and the Ca^2+^-dependent C-type lectin (*CLEC4M*) (Tan et al., 2019).

Heat shock proteins (HSPs) are a large family of chaperones (Milani et al., 2019), which are classified by their molecular weights, such as HSP27, HSP40, HSP60, HSP70, and HSP90 (Yun et al., 2019). HSPs can regulate cellular proliferation and differentiation which are strongly implicated in cancer development and progression (Chatterjee and Burns, 2017). Intriguingly, studies have verified the abnormal expression levels of HSPs in different types of cancer, including prostate, bladder, breast, ovarian, colorectal, and lung cancers (Calderwood et al., 2006;  Ledford, 2011). The overriding role of the HSPs is to stabilize the active functions of overexpressed and mutated cancer genes (Calderwood and Gong, 2016). HSPs are associated with the outcomes of anticancer therapies, such as radiotherapy and immunotherapy in lung cancer patients (Li et al., 2016; Das et al., 2019). They can facilitate protein folding and maintain protein structures that regulate cellular metabolisms, which are essential for cell survival and proliferation (Chatterjee and Burns, 2017). What’s more, HSPs have been reported to be associated with lung cancer patient’s prognosis (Liu et al., 2019). The HSPB (HSP27) can protect cells from damage induced by stress factors, and it can also regulate cell proliferation, differentiation, and apoptotic signal transduction (Wang et al., 2011). The expression of HSPB1 have distinct prognostic values in NSCLC patients, it can induce resistance to cisplatin in A549 cell through the regulation of Transforming growth factor β (TGF-β) (Huang et al., 2017; Huang et al., 2018). HSPA (HSP70) could be a valuable diagnostic and prognostic marker in lung cancer patients, and high serum HSPA level predicted unfavorable survival in SCLC patients (Balazs et al., 2017; Sojka et al., 2019). HSPB and HSPA are also very important in DNA damage and repair signaling pathway, they could be a general regulator of DNA repair, ensuring the turnover of nuclear proteins required for proper DNA repair (Dubrez et al., 2020). It has been reported that DNA damage and repair is closely relevant to cancer prognosis (Silva et al., 2019), and DNA repair genes’ polymorphisms are also found to be associated with lung cancer patient’s prognosis treated with platinum-based chemotherapy (Butkiewicz et al., 2012; Perez-Ramirez et al., 2019).

Rho GTPases, including RHO, RAC1, and CDC42, are molecular switches which can control a wide variety of signal transduction pathways in all eukaryotic cells (Reiner and Lundquist, 2018). Rho GTPases play important roles in the process of cell migration, adhesion, intracellular transport and cellular transformation (Guan et al., 2020). Rho GTPases can also regulate lung cancer cell migration and invasion by activating β-catenin signaling pathway (Wang et al., 2019). The overexpression of RAC1 was related to poor differentiation, high TNM stage, and lymph node metastasis in NSCLC patients, while down-regulation of RAC1 can reduce cell migration and invasion and sensitize cells to antitumor drugs (Chen et al., 2011). Moreover, the inhibition of RAC1 can also sensitize gefitinib-resistant NSCLC cells to gefitinib (Kaneto et al., 2014).

Genotype mutations in some key genes, including single nucleotide polymorphisms (SNPs), may cause disorder or over-activation of some specific signaling pathway, leading to tumor development and affect patients’ prognosis (Xu et al., 2019). We have found that RAC1 polymorphisms are associated with platinum-based chemotherapy toxicity in lung cancer patients in our previous study (Zou et al., 2016). In this study, we want to further explore the prognostic roles of the HSPs and Rho GTPases polymorphisms in lung cancer patients treated with platinum-based chemotherapy. The purpose of this investigation was to improve the prognosis of lung cancer patients and provide a basis for the development of lung cancer treatment measures.

## Materials and Methods

### Study Subjects and Treatment Procedures

All patients were selected by the following inclusion criteria: (1) Patients newly diagnosed with lung cancer by histological examination at the Affiliated Cancer Hospital or Xiangya Hospital of Central South University (Changsha, Hunan, China) from August 2009 to January 2013; (2) Patients should receive at least 2 periods of platinum-based chemotherapy; (3) Patients with no history of surgery before chemotherapy. The clinical characteristics of these lung cancer patients enrolled are shown in **Table 1**. All patients were provided written informed consent before they participated in this study. The study protocol was approved by the Ethics Committee of Xiangya School of Medicine, Central South University.

**Table 1:** Clinical characteristic of lung cancer patients.

Patient characteristics	N (%)
Total no. of patients	346
Gender	
Male	286(82.7)
Female	60(17.3)
Age	
≤55	154(44.5)
>55	192(55.5)
Clinical stage	
I–II	8(2.3)
III–IV	251(72.5)
LD	39(11.3)
ED	43(12.4)
Smoking status	
Non-smoker	122(35.3)
Smoker	224(64.7)
Histology	
Adenocarcinoma	112(32.4)
Squamous cell	122(35.3)
Small cell	100(28.9)
Chemotherapy regimens	
Platinum/gemcitabine	103(29.8)
Platinum/paclitaxel	60(17.3)
Platinum/navelbine	7(2.0)
Platinum/etoposide	71(20.5)
Platinum/irinotecan	6(1.7)

### Data Collection

The termination date for patient follow-up was July 15, 2019. Survival data were collected by telephone follow-up or residence registration. Overall survival (OS) time was defined as the time period between diagnosed of lung cancer and the date of the last follow-up or the death. Progression-free survival (PFS) time was calculated from the date diagnostic of lung cancer until the date of the first local recurrence or metastases in the last follow-up. Patients at the date of the last contact without progression were censored. As researchers, we were unaware of the presence of polymorphisms in the patients.

### SNP Selecting, DNA Extraction, and Genotyping

There were 19 common SNPs of *HSP*s and *Rho GTPases* selected in our study (**Table 2**). The candidate SNPs were located 5 kb upstream of the first exon and downstream of the last exon respectively. We used Haploview version 4.2 to choose the Haplotype tagging SNPs. They were chosen based on our previous research HSPs and Rho GTPases SNPs were related to lung cancer platinum-based chemotherapy toxicity (Zou et al., 2016), and they were associated with the outcome of cancers and involvement in multiple cancers (Li et al., 2016; Hung et al., 2020). And the selected SNPs must meet the condition that the minor allele frequency (MAF)>0.05 in the HapMap CHB population. The DNA we used for genotyping was separated from a 5ml peripheral blood sample using FlexiGene DNA Kit (Qiagen, Hilden, Germany). And all the samples were stored at 4°C before using. Genotyping were conducted by Sequenom’s MassARRAY system (Sequenom, San Diego, California, USA).

**Table 2:** The 19 gene polymorphisms examined in this study.

Gene	Locus	dbSNP	Call Rate(%)	Polymorphism	MAF
*HSPA4*	5q31.1	rs3088225	97.69	A/G	0.34
		rs4616886	99.13	C/T	0.21
*HSPB1*	7q11.23	rs2009836	97.40	A/G	0.22
		rs2070804	97.98	G/T	0.29
		rs2868370	97.69	C/T	0.15
		rs2868371	98.84	C/G	0.29
		rs7459185	98.84	C/G	0.43
*HSPE1*	2q33.1	rs13386066	98.55	A/G	0.21
		rs17730989	98.27	C/T	0.27
		rs2605039	98.84	G/T	0.48
*RAC1*	7p22	rs10951983	99.41	A/G	0.07
		rs12536544	97.69	A/G	0.26
		rs3813517	98.84	A/G	0.12
		rs4720672	99.13	C/T	0.17
		rs836548	98.55	A/T	0.23
		rs836554	96.24	C/T	0.28
		rs836556	99.42	C/T	0.09
*RhoA*	3p21.3	rs3811699	100.00	A/G	0.07
		rs9878943	96.53	A/G	0.06

### Statistical Analysis

We used Cox proportional hazard models to analyze the differences in age, gender, smoking status, histology, and clinical stage between the OS and PFS. We also screened the covariates used forward stepwise method of Cox proportional hazard models. Variables which were associated with OS or PFS significantly were considered as the covariates. And then, we fit these covariates into multivariate logistic regression model to adjust the covariates, through the command of –covar in PLINK. The *p* value was 2-sided and *p*<0.05 was considered statistically significant. All association analyses were conducted by three models including additive, dominant, and recessive. The additive model is for the additive effects of SNPs. It means that, if D is a minor allele and d is the major allele, the additive model means DD *versus* Dd *versus* dd. Dominant and recessive models are tests for the minor allele with two of the classes pooled. The dominant model means (DD, Dd) *versus* dd, and the recessive model means DD *versus* (Dd, dd). The aforementioned statistical analyses were performed using PLINK (ver 1.07, http://pngu.mgh.harvard.edu/purcell/plink/) and SPSS 18.0 (SPSS Inc, Chicago, Illinois, USA)

## Results

### Characteristics and Survival Status of the Lung Cancer Patients

The demographic characteristics of the 346 lung cancer patients are provided in **Table 3**. Most patients were male (82.7%), and the median age at the time of the lung cancer patients diagnosed was 55 years (range 21–77 years). The median survival time of overall survival (MST-OS) is 4.42 year, and the median survival time of progression free survival (MST-PFS) is 3.16 year. The detailed information of the associations between clinical pathology characteristics and outcomes in lung cancer patients were also summarized in **Table 3**. And the Cox proportional hazard models were analyzed to find the covariates, and the results revealed that there are no significant differences between age, gender, smoking status, histology, and clinical stage with OS or PFS as shown in **Table S1** (p>0.05).

**Table 3:** Distribution of characteristics in lung cancer patients and prognosis analysis.

Variables	Patients N (%)	Death N (%)	MST-OS (year)	MST-PFS (year)
**Lung cancer**	346	279	4.42	3.16
NSCLC	234(67.6)	189(67.7)	4.58	3.25
SCLC	100 (28.9)	80(28.7)	4.35	3.10
**Gender**				
Male	286(82.7)	229(82.1)	4.42	2.93
Female	60(17.3)	50(17.9)	4.77	4.47
**Age**				
≤55	154(44.5)	127(45.5)	4.52	3.09
>55	192(55.5)	152(54.5)	4.35	3.21
**Clinical stage**				
I/II/LD	47(13.6)	39(14.0)	3.80	3.19
III/IV/ED	294(85.0)	240(86.0)	4.53	3.16
**Smoking status**				
Non-smoker	122(35.3)	97(34.8)	4.83	4.60
Smoker	224(64.7)	182(65.2)	4.13	2.61

MST, median survival time; OS, overall survival; PFS, progression free survival; NSCLC, non-small lung cancer; SCLC, small cell lung cancer; LD, Limitation period; ED, Extensive period.

### Association Between the Polymorphisms and Prognosis in the Lung Cancer Patients

**Figure 1:**
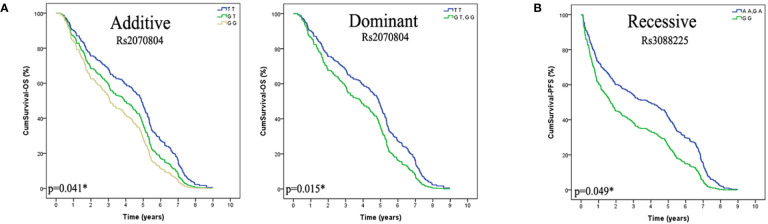
The HSPB1 rs2070804 and HSPA4 rs3088225 polymorphisms are significantly associcated with the prognosis in lung cancer patients treated with platinum-based chemotherapy, and the T variant allele of HSPB1 rs2070804, the A allele of HSPA4 rs3088225 are protective alleles. **(A)** Lung cancer patients that carried the genotype of HSPB1 rs2070804 TT have a longer overall survival time than GT or GG carries in Additive model (p=0.041*), and in the dominant model (p=0.015*). **(B)** Patients that carry the genotype of HSPA4 rs3088225 AA and GA have a longer progression free survival time than GG in Recessive model (p=0.049*).

We found that the genetic polymorphism of HSPB1 rs2070804 (G>T) was significantly associated with the overall survival (OS) of lung cancer patients in additive and dominant models [Additive model: p=0.041, OR=0.66, 95%CI, (0.45–0.98); Dominant model: p=0.015, OR=0.55, 95%CI, (0.34–0.89)]. Patients who carry the HSPB1 rs2070804 GG genotype had a significantly shorter MST-OS than the patients who have the HSPB1 rs2070804 GT or TT variant genotypes (MST-OS: 3.047, 3.942, 4.971 years, respectively). What’s more, the genetic polymorphism of HSPA4 rs3088225 (A>G) was significantly associated with the progression free survival (PFS) of lung cancer patients in recessive model [p=0.049, OR=0.44; 95%CI, (0.19–1.00)]. Which means that the patients who carry the HSPA4 rs3088225 GG variant genotype have a shorter MST-PFS than the patients who have the HSPA4 rs3088225 AA or GA genotypes (MST-PFS: 2.504, 4.219, 3.103 year, respectively). In conclusion, those patients carrying the HSPB1 T allele rs2070804 and the allele A of HSPA4 rs3088225m are carriers of the protective allele in terms of the prognosis of lung cancer (**Table 4**, **Figure 1**).

**Table 4:** Association of the HSPB1 rs2070804 polymorphisms and OS, HSPA4 rs3088225 polymorphisms and PFS in lung cancer patients.

Gene	Polymorphisms	Genotype	MST(year)	Additive		Dominant		Recessive	
				OR(95%CI)	*p* value	OR(95%CI)	*p* value	OR(95%CI)	*p* value
HSPB1	rs2070804	GG	3.047	0.66(0.45–0.98)	0.041*	0.55 (0.34–0.89)	0.015*	0.87(0.35–2.17)	0.761
		GT TT	3.942 4.971						
HSPA4	rs3088225	AA	4.219	0.77(0.55–1.09)	0.140	0.85(0.55–1.33)	0.477	0.44(0.19–1.00)	0.049*
		GA	3.103						
		GG	2.504						

*p < 0.05.

**Figure 2:**
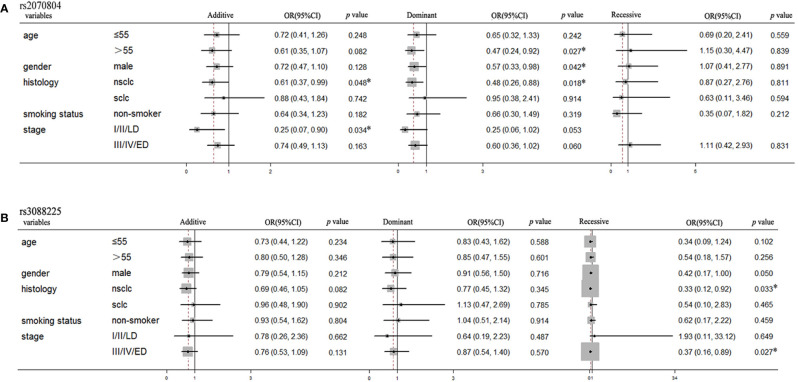
The HSPB1 rs2070804 and HSPA4 rs3088225 polymorphisms are significantly associated with the survival time in the subgroups of lung cancer patients treated with platinum-based chemotherapy. **(A)** HSPB1 rs2070804 polymorphism is significantly associated with the overall survival time in age more than 55 years, male, NSCLC, and patients whose clinical stage are I/II/LD. **(B)** HSPA4 rs3088225 polymorphism is significantly correlated with the progression free survival time in male, NSCLC, and patients whose clinical stage are III/IV/ED.

To further investigate the association between these two SNPs and the prognosis in lung cancer patients, we performed subgroup analysis based on age, gender, smoking status, histology and stage. As shown in **Figure 2**, the polymorphisms of HSPB1 rs2070804 was significantly associated with the overall survival in dominant model in age more than 55 years old [p=0.027, OR=0.47, 95%CI, (0.24–0.92)] and male patients [p=0.042, OR=0.57, 95%CI, (0.33–0.98)]. It was also correlated to the overall survival in NSCLC patients in additive [p=0.048, OR=0.61, 95%CI, (0.37–0.99)] and dominant models [p=0.018, OR=0.48, 95%CI, (0.26–0.88)], and significant with patients whose clinical stage are at I/II/LD in additive model [p=0.034, OR=0.25, 95%CI, (0.07–0.90)]. The polymorphisms of HSPA4 rs3088225 was closely related to the progression free survival in NSCLC [p=0.033, OR=0.33, 95%CI, (0.12–0.92)] and patients whose clinical stage are in III/IV/ED [p=0.027, OR=0.37, 95%CI, (0.16–0.89)] in recessive model.

### Stratification Analyses of Association Between Polymorphisms and Prognosis in Lung Cancer Patients

To further elucidate the association between the other 17 SNPs and the prognosis in lung cancer patients, we also performed subgroup analysis based on age, gender, smoking status, histology, and clinical stage. As shown in **Table 5**, the HSPE1 rs2605039 was related to overall survival in SCLC patients in dominant model [p=0.047, OR=0.39, 95%CI, (0.15–0.99)]. The RAC1 rs836548 [(Additive model: p=0.026, OR=2.01, 95%CI, (1.09–3.70); Dominant model: p=0.017, OR=2.46, 95%CI, (1.17–5.14)] and rs12536544 [Dominant model: p=0.039, OR=2.15, 95%CI, (1.04–4.43)] were significantly associated with overall survival in age less than 55-year old patients. The RAC1 rs3813517 was closely related to overall survival in patients whose clinical stage are at I/II/LD in dominant models [p=0.043, OR=5.25, 95%CI, (1.05–26.2)]. The HSPB1 rs2868370 was correlated with progression free survival in NSCLC [Additive model: p=0.012, OR=1.94, 95%CI, (1.16-3.25); Dominant model: p=0.033, OR=1.89, 95%CI, (1.05–3.37)], SCLC [Additive model: p=0.030, OR=0.30, 95%CI, (0.10–0.89); Dominant model: p=0.037, OR=0.28, 95%CI, (0.09–0.92)] and smoking patients [Additive model: p=0.021, OR=2.36, 95%CI, (1.14–4.89); Dominant model: p=0.021, OR=2.65, 95%CI, (1.16–6.06)]. The HSPB1 rs2009836 was closely associated with progression free survival in NSCLC patients in additive model [p=0.030, OR=1.61, 95%CI, (1.05–2.46)].

**Table 5:** Stratification analyses of Association between polymorphisms and OS or PFS in lung cancer patients.

OS/PFS	Gene	Polymorphisms	Subgroup	Additive		Dominant		Recessive	
				OR(95%CI)	*p* value	OR(95%CI)	*p* value	OR(95%CI)	*p* value
OS	HSPE1	rs2605039	SCLC	0.69(0.36–1.32)	0.259	0.39(0.15–0.99)	0.047*	1.28(0.40–4.14)	0.679
OS	RAC1	rs836548	age ≤ 55	2.01(1.09–3.70)	0.026*	2.46(1.17–5.14)	0.017*	1.92(0.41–8.96)	0.408
		rs12536544	age ≤ 55	1.70 (0.96–2.98)	0.067	2.15(1.04–4.43)	0.039*	1.45(0.40–5.28)	0.576
		rs3813517	I/II/LD	3.06(1.00–9.35)	0.050	5.25(1.05–26.2)	0.043*	6.27(0.58–67.4)	0.130
PFS	HSPB1	rs2868370	NSCLC	1.94(1.16–3.25)	0.012*	1.89(1.05–3.37)	0.033*	0.33(0.12–0.92)	0.033
			SCLC	0.30(0.10–0.89)	0.030*	0.28(0.09–0.92)	0.037*		
			Non-smoking	2.36(1.14–4.89)	0.021*	2.65(1.16–6.06)	0.021*	3.62(0.37–35.85)	0.271
		rs2009836	NSCLC	1.61(1.05–2.46)	0.030*	1.66(0.96–2.86)	0.069	2.68(0.94–7.64)	0.066

*p < 0.05.

## Discussion

In this study, we found that the HSPB1 rs2070804 and HSPA4 rs3088225 polymorphisms are significantly associated with the prognosis in lung cancer patients with the platinum-based chemotherapy treatment. Patients who carry the rs2070804 T variant allele are more likely to have a longer overall survival (OS) time than patients with rs2070804 G allele. These relationship are mainly reflected in male, NSCLC, age more than 55-year-old patients and patients whose clinical stage are in I/II/LD. Moreover, the progression free survival (PFS) time in patients who carry the rs3088225 G variant allele are more likely to be shorter than patients with rs3088225 A allele. The rs3088225 polymorphisms in the subgroups of male, NSCLC, and clinical stage in III/IV/ED patients are related to the PFS time significantly in the stratified analysis. In conclusion, the HSPB1 rs2070804 T allele and HSPA4 rs3088225 A allele are the protective allele in the prognosis of lung cancer patients treated with platinum-based chemotherapy.

It has been reported that the expression of heat-shock proteins (HSPs) may be prognostic markers in several tumor types through the regulation of cell proliferation, invasion and metastasis (Saini and Sharma, 2018). And the inhibition of HSPs is currently an attractive potential therapeutic approach against cancer (Kaigorodova and Bogatyuk, 2014). HSPB suppression can result in the apoptotic death of MET-addicted EBC-1 in lung cancer cells, and oncogene-addicted cells require HSPB for survival (Konda et al., 2017). Abnormalities in the transforming growth factor b (TGF.B) pathway, is widely observed in drug resistance during lung cancer chemotherapy (Yokokura et al., 2016). HSPB can induce the resistance to cis-platinum in A549 cells through the regulation of TGF-β *via* decreasing cell viability and increasing cell apoptosis in A549 cell (Huang et al., 2017). HSPB1 can inhibit the endothelial-to-mesenchymal transition (EDMT) to suppress pulmonary fibrosis and lung tumorigenesis (Choi et al., 2016). It has been reported that the increased HSPB expression was correlated with malignant biological behavior of NSCLC and the increased HSPB expression was also related to the shorter survival of NSCLC patients (Sheng et al., 2017). There are some variants of HSPB found to be correlated with its expression level. The functional HSPB1 promoter -1271G>C variant may affect lung cancer susceptibility and survival time by modulating endogenous HSPB synthesis levels (Guo et al., 2010). The SNPs of HSPB1 rs7459185 was associated with radiation esophagitis in lung cancer through the regulation of HSPB1 expression level (Delgado et al., 2019). HSPB can regulate aggressive tumor behavior, metastasis, poor prognosis, and resistance to chemotherapy through the interference with theses effectiveness of targeted agents (Choi et al., 2017). There was one report about HSPB polymorphism found to be associated with NSCLC prognosis, in the US patients, it found that the CC genotype of HSPB1 rs2868371 was associated with poorer overall survival in US patients with NSCLC after radio(chemo)therapy (Xu et al., 2012). HSPA expression is a powerful and significant prognostic indicator in NSCLC patients, which is related to histopathological differentiation, lymph node metastasis, patients’ clinical stages, and smoking history (Huang et al., 2005). HSPA was reported to be a positive predictive factor in completely resected NSCLC treated with platinum-based adjuvant chemotherapy (Park et al., 2014). It was interestingly observed that HSPA1 was associated with good prognosis while HSPA2 correlated with bad prognosis in primary NSCLC (Sojka et al., 2019). HSPA1B A(1267)G polymorphism can influence the HSPA1B expression of SCLC cells, and the expression of HSPA1B was significantly decreased in GG as compared to cells of AA or AG genotype patients. The variant of GG genotype may be a negative prognostic factor for survival in SCLC patients, as the survival time of patients carry the GG genotype was significantly shorter as compared to carriers of the A allele (Szondy et al., 2012). The functional HSPA1B rs2763979 and rs6457452 variants are associated with lung cancer risk and survival (Guo et al., 2011). However, there are no reports about the relationship of HSPs polymorphisms and prognosis in platinum-based chemotherapy treatment of Chinese lung cancer patients.

The three GTPases, RHO, RAC, and Cdc42, play important roles in harmonizing many cellular activities among embryonic development, both in healthy cells and in disease conditions like cancers, RAC1 is significantly associated with the cell proliferation, metastasis-associated phenotypes, and drug-resistance especially on solid tumors (De et al., 2019). It has been reported that the overexpression of RAC1 is related to the poor prognosis and metastasis in NSCLC through the regulation of Epithelial Mesenchymal Transition, which means that RAC1 may be a potential therapeutic target in the treatment of NSCLC patients (Zhou et al., 2016). The Rho kinase pathway has an intimate relationship with cell growth, cell migration, and invasion in lung cancer, it has been found that RhoA knockdown can prevent cell proliferation and induces apoptosis in SPCA1 lung cancer cells (Liu et al., 2017). It has been reported that RAC1 or RhoA are mutated in cancers, and the expression levels of RAC1 or RhoA are aslo altered, which means that RhoA may play an crucial role in lung cancer treatment (Haga and Ridley, 2016).

Our study investigated the association between HSPs, RAC1 and RhoA and the survival time in Chinese lung cancer patients treated with platinum-based chemotherapy. All the patients we enrolled received the platinum-based chemotherapy regimens treatment for at least two periods, and we also performed stratified analysis in age, gender, smoking status, histology and clinical stage. Except for the polymorphisms of HSPB1 rs2070804 and HSPA4 rs3088225, we also found other polymorphisms which are associated with lung cancer prognosis in some specific subgroups. For instance, HSPE1 rs2605039 was correlated to the overall survival in SCLC patients (p=0.047). RAC1 rs836548 and rs12536544 were associated with the overall survival in age less than 55 years old patients (p=0.017, p=0.039, respectively), and RAC1 rs3813517 was related to overall survival in patients whose clinical stage were in I/II/LD (p=0.043). HSPB1 rs2868370 was associated with the progression free survival in NSCLC, CLCL and non-smoking patients (p=0.012, p=0.030, p=0.021, respectively). HSPB1 rs2009836 was also correlated to the progression free survival in NSCLC patients (p=0.030).

However, there were several limitations in our study. First, the sample size was not large enough, we just enrolled 346 patients in our study. Second, the biological function mechanisms of these SNPs need further study *in vitro*. Finally, the validation of our results needs replication studies with other independent subjects.

In conclusion, we found that the polymorphisms of HSPB1 rs2070804 and HSPA4 rs3088225 were significantly associated with the prognosis in lung cancer patients treated with platinum-based chemotherapy. Lung cancer patients who carry the HSPB1 rs2070804 T allele or HSPA4 rs3088225 A allele may have a better prognosis compared to the rs2070804 G allele or HSPA4 rs3088225 G allele. The genotypes of HSPB1 rs2070804 and HSPA4 rs3088225 may be an attractive biomarker used to predict the prognosis of platinum-based chemotherapy lung cancer patients.

## Data Availability Statement

The raw data supporting the conclusions of this article will be made available by the authors, without undue reservation.

## Ethics Statement

The studies involving human participants were reviewed and approved by Ethics Committee of Xiangya School of Medicine, Central South University. The patients/participants provided their written informed consent to participate in this study.

## Author Contributions

Study design was contributed by TZ, J-YL, JC, and Z-QL, with assistance from the rest of the authors. TZ and LS took the lead in sample collection and data analysis, assisted by J-YY, XL, X-PL, and H-HZ. Data interpretation was performed by TZ, with assistance from the other authors. The manuscript was written primarily by TZ and JC, with assistance from the other authors, and revised by Z-QL. All authors contributed to the article and approved the submitted version.

## Conflict of Interest

The authors declare that the research was conducted in the absence of any commercial or financial relationships that could be construed as a potential conflict of interest.
