# Supplementary material for: The Association Between Heat-Shock Protein Polymorphisms and Prognosis in Lung Cancer Patients Treated With Platinum-Based Chemotherapy
Source: Front Pharmacol. 2020 Jul 21;11:1029. doi: 10.3389/fphar.2020.01029 (PMC7396685; doi:10.3389/fphar.2020.01029)
Supplement: Supplementary file 1 [file Table_1.docx]

Table S1 COX regression analysis of the prognosis of OS and PFS in lung cancer patients

| Variables | β | | SE | | OR | | 95%CI | | | P | |
| --- | --- | --- | --- | --- | --- | --- | --- | --- | --- | --- | --- |
|  | OS | PFS | OS | PFS | OS | PFS | OS | PFS | | OS | PFS |
| Age | -0.010 | -0.111 | 0.128 | 0.115 | 0.990 | 0.895 | 0.771-1.273 | 0.714-1.121 | | 0.940 | 0.334 |
| Gender | -0.238 | -0.191 | 0.213 | 0.190 | 0.788 | 0.826 | 0.519-1.197 | 0.569-1.199 | | 0.264 | 0.826 |
| Smoking statues | -0.074 | -0.176 | 0.173 | 0.153 | 0.929 | 0.838 | 0.662-1.305 | | 0.622-1.131 | 0.671 | 0.838 |
| Histology | -0.019 | -0.064 | 0.166 | 0.142 | 0.981 | 0.938 | 0.709-1.357 | | 0.710-1.239 | 0.908 | 0.652 |
| stage | 0.133 | 0.060 | 0.215 | 0.185 | 1.142 | 1.062 | 0.749-1.741 | | 0.740-1.526 | 0.537 | 0.743 |
